# EHMTI-0231. Headache as primary symptom in the neurological emergency department – epidemiological data analysis

**DOI:** 10.1186/1129-2377-15-S1-D37

**Published:** 2014-09-18

**Authors:** A Liverezas, I Stavropoulos, D Athanasopoulos, S Tsiara

**Affiliations:** 1 Neurology, General Hospital of Athens "G.Gennimatas", Athens, Greece

## Introduction

Headache is one of the most common neurological symptoms that lead patients to the ED. The latest data show that 50-75% of adults globally, have experienced headache at least once during the past year and about 1-4% can be affected for several days per month, resulting in a huge disease load that increases the cost of health care worldwide.

## Aim

To present data regarding patients that visited the Neurological Emergency department (NED) of General Hospital of Athens “GNA G. Gennimatas” with headache as primary complaint and analyze the demographic, qualitative and quantitative characteristics of them.

## Method

We analysed the data of the patients that visited the NED from 1/12/2012 to 1/10/2013. Data analysis was based on age, sex, medical history, type of headache, management, red flags and cost. Patients with more major complaints or insufficient data were excluded.

## Results

8045 patients were examined and 1232 presented with headache (15.3%). 63.9% were female and this percentage was similar in all age groups. The most common primary headaches were migraine followed closely by tension headache. The prevalence of secondary headaches was 15.9% and 32.65% of these had red flags. The total cost of the basic laboratory and imaging investigations is estimated 67653.4 euros.

## Conclusions

The majority of headaches in NED are primary. Red flags are important risk factors for secondary headaches. The clinicians ability to recognize the headache type could reduce the economical cost.

No conflict of interest.

